# Diversity, community composition, and antibacterial activity of endophytic fungi isolated from two *Gynostemma* species

**DOI:** 10.3389/fmicb.2026.1867726

**Published:** 2026-08-24

**Authors:** Ruixi Zhou, Jingwei Zhang, Guanqun Zhan, Zengjun Guo

**Affiliations:** School of Pharmacy, Health Science Center, Xi’an Jiaotong University, Xi’an, China

**Keywords:** antibacterial activity, community structure, endophytic fungi, fungal taxonomy, *Gynostemma longipes*, *Gynostemma pentaphyllum*

## Abstract

Endophytic fungi are recognized as important sources of bioactive natural products, yet the extent to which host plant identity and chemistry shape their community assembly and functional potential remains insufficiently understood. To address this, we comparatively investigated the diversity, community composition, and antibacterial activity of endophytic fungi isolated from two co-cultivated medicinal plants with distinct saponin and flavonoid profiles–*Gynostemma pentaphyllum* and *Gynostemma longipes*. A total of 179 fungal isolates were obtained from five tissue types and identified using internal transcribed spacer sequencing, representing 50 taxa. Diversity analysis revealed that *G. pentaphyllum* harbored higher species richness and isolation rates than *G. longipes*. Antibacterial activity of fungal extracts tested separately against *Escherichia coli*, *Staphylococcus aureus*, and *Pseudomonas aeruginosa* was highly strain-dependent, with *S. aureus* showing the highest susceptibility. Notably, *Fusarium* sp. GYP-36 exhibited strong broad-spectrum activity, producing inhibition zones up to 25.5 mm against *E. coli* when tested with 10 μL of 1 mg mL^–1^ extract, and displaying a minimum inhibitory concentration of 64 μg mL^–1^ against both *E. coli* and *S. aureus*. Multivariate analyses indicated that fungal taxonomic identity was significantly associated with antibacterial activity, whereas host species and tissue origin showed statistically non-significant effects on the antibacterial activity of fungal isolates. Collectively, these findings demonstrate that functional bioactivity is predominantly governed by fungal taxonomy, while host-associated chemical traits play a secondary but significant role in shaping community assembly. Furthermore, this study highlights *Gynostemma*-associated endophytes as promising reservoirs for the discovery of novel antimicrobial agents, particularly through the untapped potential of taxa such as *Fusarium* sp. GYP-36.

## 1 Introduction

Infectious diseases caused by pathogenic bacteria remain a major threat to human health worldwide (GBD 2019 Diseases and Injuries Collaborators, 2020). Although antibiotics are widely used, their long-term application raises concerns about adverse effects and environmental risks (Fatima et al., 2025). The urgent need for novel antimicrobial agents has intensified the search for natural products from underexplored sources (Mussai et al., 2023; Rakhal et al., 2025; Wong Chin et al., 2021).

Endophytic fungi, which colonize plant tissues asymptomatically, have attracted increasing attention as a promising source of natural products (Sadeer et al., 2020; Varghese et al., 2024). They are ubiquitous and associated with nearly all plant species sampled, exhibiting diverse ecological associations and remarkable biosynthetic potential. Consequently, endophytic fungi are recognized as valuable sources of bioactive secondary metabolites, including antimicrobial, anticancer, and antioxidant compounds (Jeewon and Dissanayake, 2019; Liao et al., 2025; Sarma and Jeewon, 2019).

Medicinal plants are important reservoirs of bioactive compounds, but the accumulation of these compounds is not solely governed by plant genetics–it is also profoundly influenced by associated endophytic fungi. Endophytes can modulate host secondary metabolism and even produce metabolites similar to those of their hosts (Alam et al., 2021; Rampadarath et al., 2018). Thus, understanding medicinal plant resources requires integrating plant metabolite profiling with endophytic community analysis.

*Gynostemma pentaphyllum* (Cucurbitaceae) is a well-known medicinal and edible plant in China, rich in saponins, flavonoids, and polysaccharides with anti-inflammatory, antioxidant, and immunomodulatory activities (Razmovski-Naumovski et al., 2005; Li et al., 2025). Its closely related species, *G. longipes*, exhibits distinct morphological and phytochemical characteristics. Specifically, these two species differ in several morphological traits, including stem indumentum, leaflet number, leaf shape, texture, thickness, and tendril morphology. In addition, they exhibit distinct phytochemical profiles, with differences in both the abundance of major chemical constituents and the structural diversity of dammarane-type saponins (gypenosides) (Wu and Chen, 1983; Zhang et al., 2006; Chen et al., 2023; Liang et al., 2025). Therefore, these two closely related but chemically and morphologically distinct *Gynostemma* species cultivated in Pingli County, Shaanxi Province, provide a suitable comparative system for investigating the association between host characteristics and endophytic fungal community variation. Their co-cultivation within the same region under comparable environmental conditions further helps minimize potential influences from geographical and environmental differences.

Previous studies on *Gynostemma*-associated endophytic fungi have mainly focused on fungal isolation, taxonomic characterization, and the evaluation of the bioactive potential of individual strains (Fan, 2013; Ma, 2014; Ma et al., 2015). For example, Fan (2013) and Ma et al. (2015) reported diverse culturable endophytic fungi isolated from different *Gynostemma* resources, providing important information on the diversity and composition of endophytic fungal communities associated with this medicinal plant (Fan, 2013; Ma, 2014; Ma et al., 2015). Beyond their diversity, functional studies have also revealed the potential of *Gynostemma*-associated endophytic fungi. An endophytic *Aspergillus niger* isolated from *G. pentaphyllum* was reported to transform gypenosides into bioactive ginsenoside F2, demonstrating the metabolic transformation capability of these endophytic fungi (Zhang et al., 2023). Furthermore, Wang et al. isolated an endophytic fungus, *Chaetomium* sp. JY25, from *G. pentaphyllum* and investigated its fermentation conditions, polysaccharide production, and antioxidant, antibacterial, and anti-inflammatory activities, highlighting the bioactive potential of individual endophytic fungal strains (Zhang et al., 2017; Wang et al., 2019, 2021). Despite these advances, the diversity, community structure, and functional potential of endophytic fungi associated with different *Gynostemma* species remain insufficiently explored. In particular, systematic comparisons between *G. pentaphyllum* and *G. longipes* regarding their endophytic fungal assemblages and associated antibacterial activities remain limited.

To further explore these questions, this study aimed to systematically compare the diversity and community composition of culturable endophytic fungi associated with the two *Gynostemma* species. In addition, by integrating host phytochemical profiling with antibacterial activity evaluation of fungal isolates, this study further evaluated the potential associations between fungal functional activity and host chemical characteristics, fungal taxonomic composition, and tissue origin. These findings provide insights into the relationships between medicinal plants and their associated endophytic fungi, contributing to a better understanding of plant–endophyte interactions and the functional potential of medicinal plant-associated endophytic fungi.

## 2 Materials and methods

### 2.1 Plant materials and sampling

Healthy samples of *G. pentaphyllum* and *G. longipes* were collected from Pingli County, Ankang City, Shaanxi Province, China in July 2023. The plant species were identified by Prof. Zengjun Guo (School of Pharmacy, Xi’an Jiaotong University) based on morphological characteristics described in the Flora of China. Representative plant samples are retained in the laboratory under internal accession numbers GYP-01 (for *G. pentaphyllum*) and GYP-02 (for *G. longipes*). Morphological characteristics of the two species are summarized in Supplementary Table 1.

### 2.2 Reagents

All commercial chemicals and solvents were of analytical grade. Ginsenoside Rb_1_ (≥98%, HPLC) and rutin (≥98%, HPLC) were purchased from Chengdu Pusi Biotechnology Co., Ltd., (Chengdu, China). Penicillin sodium (≥96.0%), vancomycin hydrochloride (USP, ≥900 μg mL^–1^), gentamicin sulfate (USP) and DMSO (≥99.9%) were supplied by Shanghai Aladdin Biochemical Technology Co., Ltd. Ultrapure water (Wahaha pure water, Hangzhou Wahaha Group Co., Ltd., Hangzhou, China) was used throughout the experiments.

### 2.3 Quantification of plant active constituents

Saponins, flavonoids, and polysaccharides, as the major bioactive constituents of *Gynostemma* species, were quantified by UV–Vis spectrophotometry. Total saponins were quantified using the vanillin–perchloric acid colorimetric method. Samples were subjected to ultrasonic extraction with methanol and filtered. The extracts were subsequently reacted with vanillin solution and perchloric acid at 60°C for 15 min, followed by rapid cooling in an ice bath. After the addition of acetic acid, the absorbance was measured at 550 nm. Quantitative analysis was performed using a calibration curve constructed with ginsenoside Rb_1_ as the reference standard (Hiai et al., 1976). Total flavonoids were quantified using the aluminum nitrate colorimetric method. Samples were subjected to ultrasonic extraction with 65% ethanol and filtered. The extracts were sequentially reacted with sodium nitrite, aluminum nitrate, and sodium hydroxide under controlled conditions. The absorbance was measured at 510 nm. Quantification was carried out using a calibration curve constructed with rutin as the reference standard (Chang et al., 2002). Total polysaccharides were quantified using the phenol–sulfuric acid method. Samples were subjected to ultrasonic extraction with water and filtered. The extracts were reacted with phenol and concentrated sulfuric acid, followed by incubation at room temperature for 15 min. The absorbance was measured at 625 nm. Quantitative determination was performed using a calibration curve constructed with D-glucose reference standard (DuBois et al., 1956). Calibration curves were established using a series of standard solutions, and all measurements were conducted in triplicate. Detailed colorimetric conditions are provided in Supplementary Table 2.

### 2.4 Isolation of endophytic fungi

Fresh and healthy *Gynostemma* plants were collected, and five tissue types, including main roots, fibrous roots, stems, leaves, and shoot tips (Supplementary Figure 1), were aseptically excised in a laminar flow hood. To remove epiphytic microorganisms, the collected tissues were surface sterilized following previously described protocols for endophytic fungal isolation (Schulz et al., 1993; Arnold et al., 2001). Briefly, plant tissues were sequentially treated with 75% ethanol and sodium hypochlorite solution, followed by repeated rinsing with sterile distilled water. To verify the effectiveness of surface sterilization, the final rinse water was inoculated onto PDA plates as a sterility control and incubated under the same conditions. No microbial growth was observed after incubation, confirming the effectiveness of the sterilization procedure. The sterilized tissues were then cut into small segments and used for endophytic fungal isolation using a tissue segment method (Schulz et al., 1993).

The tissue segments were inoculated onto PDA supplemented with penicillin (100 μg mL^–1^) and streptomycin (100 μg mL^–1^), as well as high-salt Czapek medium. For each tissue type, three independent biological replicates were prepared, with three technical replicates per medium. All plates were sealed with parafilm and incubated in an inverted position at 28°C. Fungal growth was examined at 12 h intervals. Newly emerging fungal growth was aseptically transferred onto fresh plates and further incubated at 28°C for 5–7 days. Pure isolates were obtained after repeated subculturing.

During the isolation process, fungal growth emerging from each tissue segment was carefully examined for seven consecutive days. The positions of newly emerging fungal growth were marked on the back of the plates, and fungal growth with distinct colony morphology, pigmentation, growth characteristics, or emergence positions was separately transferred for purification. Colonies appearing during later incubation stages were also collected to improve the recovery of slow-growing or less competitive endophytic fungi. For isolates with similar colony morphology, microscopic observation and ITS sequence identification were further performed, and potential duplicate isolates were excluded based on morphological characteristics and molecular identification results.

### 2.5 Identification of fungal isolates

Genomic DNA was extracted from fungal mycelia using the E.Z.N.A.^®^ HP Fungal DNA Kit (Omega Bio-tek, Norcross, GA, USA) according to the manufacturer’s instructions. The internal transcribed spacer (ITS) region was amplified using the universal primers ITS1 (5′-TCCGTAGGTGAACCTGCGG-3′) and ITS4 (5′-TCCTCCGCTTATTGATATGC-3′) (White et al., 1990).

PCR amplification was performed in a 25 μL reaction mixture containing 2 μL template DNA, 1 μL of each primer, 12.5 μL premix, and 8.5 μL sterile ddH2O. The thermal cycling conditions were as follows: initial denaturation at 94°C for 5 min; 25 cycles of denaturation at 94°C for 30 s, annealing at 55°C for 1 min, and extension at 72°C for 1 min; followed by a final extension at 72°C for 5 min.

PCR products were sequenced using the same primer pairs (Genewiz Biotechnology Co., Ltd., Suzhou, China). The obtained ITS sequences were compared with reference sequences in the NCBI database using the BLAST (Altschul et al., 1990) algorithm. Multiple sequence alignment was performed using MEGA version 11, and a phylogenetic tree was constructed (Kumar et al., 2018) based on the Neighbor-Joining method. Taxonomic identification was assigned based on ITS sequence analysis, including BLAST comparison with reference sequences in the NCBI database, sequence similarity evaluation, and phylogenetic placement (Jeewon and Hyde, 2016; Raja et al., 2017). Isolates were conservatively assigned at the genus level when ITS sequences did not provide sufficient resolution for reliable species-level identification.

### 2.6 Preparation of fungal extracts

Endophytic fungi were cultured on potato dextrose agar (PDA) plates at 28°C and 60% relative humidity for 4–6 days until reaching the exponential growth phase. Under aseptic conditions, agar plugs (0.5 cm × 0.5 cm) containing actively growing mycelia were excised and transferred into flasks containing sterilized rice medium (Akpotu et al., 2017). Static fermentation was conducted at 20–28°C under 50%–60% relative humidity and natural light for 2 weeks. The fermented material was then extracted with methanol by maceration. Methanol was selected as the extraction solvent because of its good penetration ability and broad extraction efficiency for diverse fungal secondary metabolites, and it has been widely used for preparing crude fungal extracts for natural product discovery and bioactivity screening (Smedsgaard, 1997; de Barros et al., 2011). The extract was filtered and concentrated under reduced pressure to yield the crude extract.

### 2.7 Antibacterial activity assay

Antibacterial activity was evaluated using the agar disk diffusion method (Bauer et al., 1966) as previously described. The standard reference strains, including *S. aureus* ATCC 25923, *P. aeruginosa* ATCC 27853, and *E. coli* ATCC 25922, were kindly provided by Prof. Jiye Zhang’s research group from the S, and were routinely maintained in our laboratory for antibacterial assays. The strains were individually inoculated into LB broth and incubated at 37°C with shaking at 180 rpm for 18 h to reach the logarithmic growth phase. The bacterial suspensions were adjusted to match the turbidity of a 0.5 McFarland standard immediately before inoculation onto agar plates.

A sterile glass spreader was used to evenly distribute 100 μL of the bacterial suspension onto Mueller-Hinton agar (MHA) plates, resulting in a confluent bacterial lawn. Sterile 6 mm filter paper disks were impregnated with 10, 15, and 20 μL of fungal extracts [prepared at a concentration of 1 mg mL^–1^ and dissolved in dimethyl sulfoxide (DMSO)]. Disks loaded with the corresponding solvents were used as negative controls. Disks loaded with penicillin sodium (10 μg/disk for *E. coli*), vancomycin hydrochloride (30 μg/disk for *S. aureus*), and gentamicin sulfate (10 μg/disk for *P. aeruginosa*) were used as positive controls. The disks were then placed onto the inoculated agar plates and incubated at 37°C for 16–20 h. The diameters of the inhibition zones were measured, and the results were evaluated according to the Clinical and Laboratory Standards Institute (CLSI) guidelines for antimicrobial susceptibility testing (CLSI, 2024). All assays were performed in triplicate, and mean inhibition zone diameters were recorded.

### 2.8 Minimum inhibitory concentration (MIC) assay

Based on the results of the agar disk diffusion assay, representative endophytic fungal isolates showing moderate to strong antibacterial activity were selected for MIC determination. MICs were determined using the broth microdilution method following CLSI M07 procedures (CLSI, 2024).

Briefly, the fungal crude extracts were dissolved in DMSO and serially two-fold diluted in sterile 96-well microplates with LB broth to achieve final concentrations ranging from 128 to 0.25 μg mL^–1^. Overnight bacterial cultures (*E. coli*, *P. aeruginosa* and *S. aureus*) were adjusted to 0.5 McFarland standard (approximately 1.5 × 10^8^ CFU mL^–1^) and further diluted 1:100 in LB broth to obtain a final inoculum of approximately 1.5 × 10^6^ CFU mL^–1^. Each well received 50 μL of the bacterial suspension and 50 μL of the serially diluted extract, resulting in a total volume of 100 μL. The final concentration of DMSO in each well did not exceed 1% (v/v), which was confirmed to have no inhibitory effect on bacterial growth. A negative control (broth only), a solvent control (DMSO), and positive controls (penicillin sodium, vancomycin hydrochloride, and gentamicin sulfate) were included. The final concentrations of each positive control antibiotic were prepared by two-fold serial dilution, ranging from 128 to 0.25 μg mL^–1^. The plates were incubated at 37°C for 18–20 h. The MIC was defined as the lowest concentration of the extract that completely inhibited visible bacterial growth. Each assay was performed in triplicate.

### 2.9 Statistical analysis

The composition and distribution of endophytic fungal assemblages were quantitatively assessed using colonization rate (CR) and isolation rate (IR). Species richness (S) was determined as the total number of taxa recorded per sample. Community diversity and dominance were evaluated using the Shannon–Wiener index (*H*′) and Simpson’s index (*D*), respectively, while evenness was quantified using Pielou’s index (*J*), which reflects the distributional uniformity of taxa. The formulas and parameter definitions for these ecological indices are provided in Supplementary Note 1 (Shannon, 1948; Simpson, 1949; Pielou, 1966).

Statistical analyses were conducted using SPSS (version 26.0). Data are presented as the mean values, with standard deviation calculated for experiments with biological replicates. Differences in CR, IR, S, and diversity indices between the two species were assessed using independent samples *t*-test. Statistical significance was defined at *P* < 0.05.

Antibacterial activity was quantified based on inhibition zone diameters, and the effects of fungal strains and application volumes were evaluated using ANOVA. Principal component analysis (PCA) (Jolliffe, 2002) was applied to examine the relationships among antibacterial performance and influencing factors, including host species, tissue origin, and fungal taxonomic identity. Hierarchical clustering analysis and heatmap visualization were conducted based on standardized inhibition zone data to characterize patterns of antibacterial activity across strains and treatment conditions. In addition, the top 10 isolates were ranked according to their overall antibacterial performance to identify strains with the highest bioactivity potential.

## 3 Results

### 3.1 Quantification of major bioactive components in two *Gynostemma* species

The contents of total saponins, polysaccharides, and flavonoids in *G. pentaphyllum* and *G. longipes* were quantified by UV–Vis spectrophotometry (Supplementary Table 3).

Total saponin content was significantly higher in *G. pentaphyllum* (12.0%) than in *G. longipes* (4.50%) (*P* < 0.05), representing an approximately 2.67-fold increase. In contrast, flavonoid content was significantly higher in *G. longipes* (3.82%) than in *G. pentaphyllum* (2.35%) (*P* < 0.05). Polysaccharide levels were comparable between the two species, with values of 6.89% and 6.70%, respectively.

At the tissue level, saponins were predominantly accumulated in leaves and stems of *G. pentaphyllum*, whereas flavonoids showed higher levels in the leaves of *G. longipes* Polysaccharides were relatively enriched in fibrous roots and shoot apices in both species.

### 3.2 Diversity and abundance of endophytic fungal communities

A total of 179 endophytic fungal isolates were obtained from five plant tissues (main roots, fibrous roots, stems, leaves, and shoot apices) of two *Gynostemma* species, with 102 isolates from *G. pentaphyllum* and 77 from *G. longipes*. These isolates were classified into 50 taxa using ITS sequence data (Supplementary Table 4). The ITS sequences obtained in this study have been deposited in GenBank (NCBI) under accession numbers PZ285361–PZ285409. The phylogenetic relationships of the isolated endophytic fungi were inferred based on ITS sequences, as shown in Supplementary Figure 2.

The colonization rate, isolation rate, and species richness of endophytic fungi in the two *Gynostemma* species are summarized in Table 1. *Gynostemma pentaphyllum* exhibited higher colonization rate (CR: 0.7111 vs. 0.5666), isolation rate (IR: 1.1333 vs. 0.8555), and species richness (S: 27 vs. 23) compared to *G. longipes*, suggesting a greater capacity to host diverse endophytic fungi. S, CR and IR differed significantly between *G. pentaphyllum* and *G. longipes* (*P* < 0.05), with *G. pentaphyllum* showing higher values. Diversity indices showed slightly higher diversity in *G. pentaphyllum*, as indicated by the Shannon index (*H*′: 3.5744 vs. 3.5014) and Simpson index (*D*: 0.9026 vs. 0.8998), these differences were not statistically significant (*P* > 0.05). In contrast, Pielou’s Evenness Index (*J*) showed comparable values between the two species, indicating similarly high species evenness and a stable distribution of endophytic fungal communities.

**TABLE 1:** Colonization rate, isolation rate and species richness of endophytic fungi in two *Gynostemma* species.

Parameters	*G. pentaphyllum*	*G. longipes*
Number of fragments incubated	90	90
Number of fragments colonized	64	51
Total number of isolates	102	77
Colonization rate (CR)	0.7111^a^	0.5666^b^
Isolation rate (IR)	1.1333^a^	0.8555^b^
Species richness (S)	27^a^	23^b^
Shannon diversity index (*H*′)	3.5744	3.5014
Simpson’s diversity index (*D*)	0.9026	0.8998
Pielou’s Evenness Index (*J*)	0.9388	0.9462

Data represent the mean of three replicates. Superscript letters represent statistically significant differences at *P* < 0.05 (for CR and IR).

### 3.3 Community composition of endophytic fungal assemblages

**FIGURE 1:**
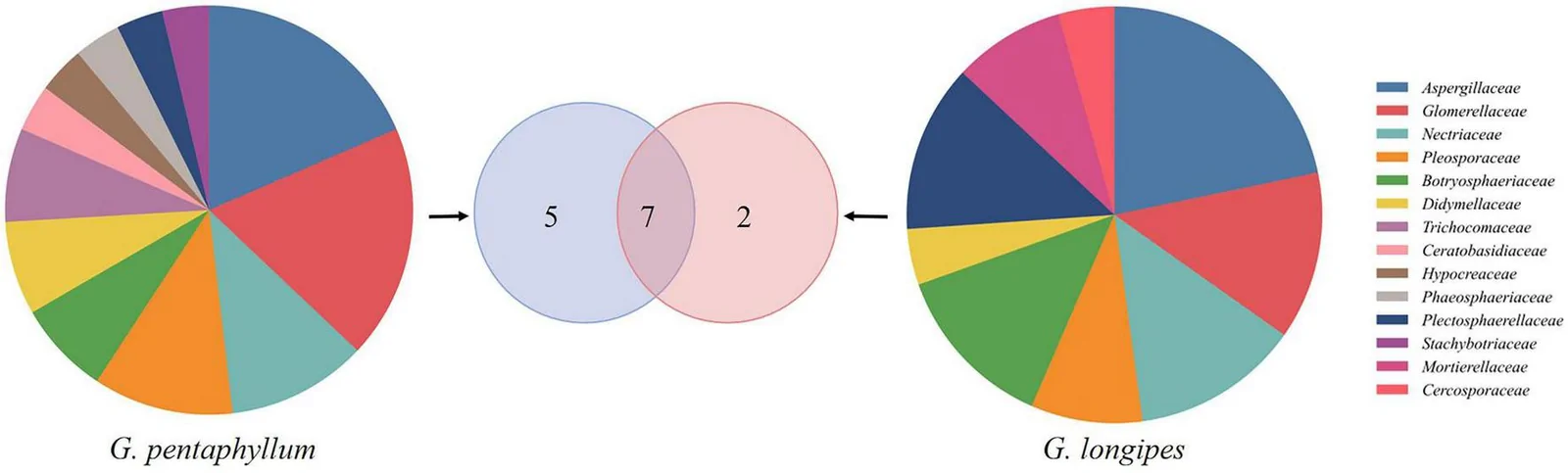
Pie charts showing the relative abundance of dominant culturable endophytic fungi in *G. pentaphyllum* and *G. longipes*. Venn’s diagram showing the unique and shared genera of culturable endophytic fungi between the two species.

**FIGURE 2:**
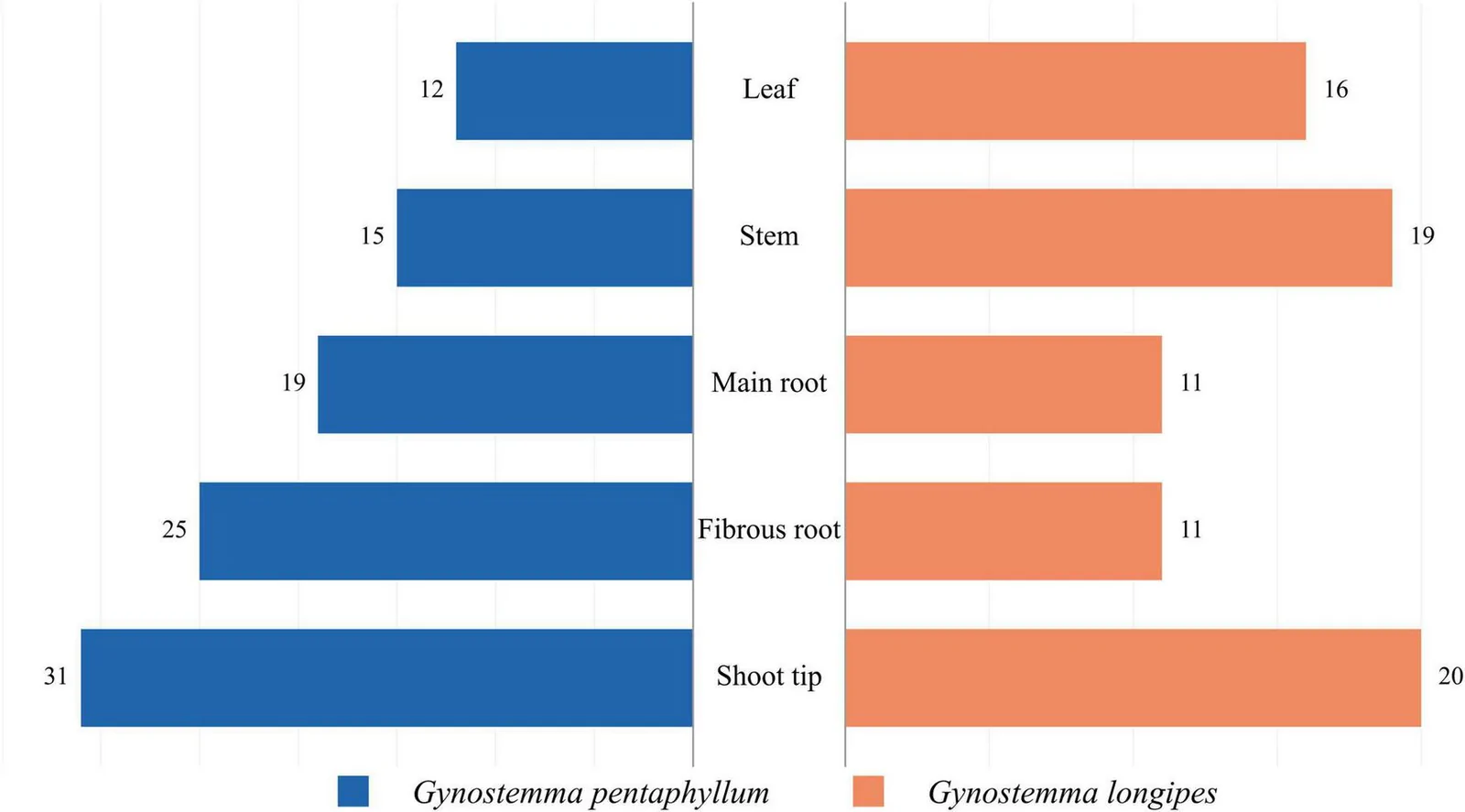
Comparison of endophytic fungal genus counts in different tissues of *Gynostemma* species. The vertical axis indicates different plant tissues, the numbers on the bars show the number of isolated fungal genera, and the bar color distinguishes the two host species: blue for *G. pentaphyllum* and orange for *G. longipes*. Shoot tips of both species harbored the highest abundance of endophytic fungi.

A total of 14 fungal families and 40 fungal genera were recovered from the two *Gynostemma* species, and significant differences were observed in the endophytic fungal community structures between the two hosts (Figure 1). Seven fungal families (Aspergillaceae, Glomerellaceae, Nectriaceae, Plectosphaerellaceae, Botryosphaeriaceae, Pleosporaceae, Didymellaceae) were shared between the two hosts, whereas five families were exclusively detected in *G. pentaphyllum* (Trichocomaceae, Hypocreaceae, Phaeosphaeriaceae, Stachybotryaceae, and Ceratobasidiaceae), and two families were unique to *G. longipes* (Mortierellaceae and Cercosporaceae). A total of 27 fungal genera were recovered from *G. pentaphyllum*, with *Alternaria* being the dominant genus (RA = 15.6%), whereas 23 genera were recovered from *G. longipes*, where *Aspergillus* was dominant (RA = 21.6%). These results indicate a higher fungal richness in *G. pentaphyllum* and a stronger dominance of *Aspergillus* in the endophytic fungal community of *G. longipes*. The shoot apices harbored the highest number of fungal genera in both hosts (Figure 2), suggesting that endophytic fungi may preferentially colonize metabolically active tissues.

### 3.4 Antibacterial activity of endophytic fungal extracts

The antibacterial activities of endophytic fungal crude extracts against *E. coli*, *S. aureus*, and *P. aeruginosa* were evaluated using the agar disk diffusion method (Supplementary Table 5). No inhibition was observed for solvent controls.

The inhibition zone diameters varied markedly among fungal isolates and target bacteria, ranging from no detectable activity (6 mm) to strong inhibition exceeding 30 mm. A clear dose-dependent pattern was observed, with inhibition zones generally increasing as the administration volume increased from 10 to 20 μL. Statistical comparisons among application volumes (10, 15, and 20 μL) were performed using one-way ANOVA. Significant differences were observed between application volumes (*p* < 0.05).

Differences in bacterial susceptibility were evident. *Staphylococcus aureus* showed the highest sensitivity, with many isolates exhibiting moderate to strong inhibition across all application volumes. *Escherichia coli* displayed intermediate susceptibility, with pronounced inhibition limited to a subset of isolates at higher application volumes. In contrast, *P. aeruginosa* was the least sensitive, with most isolates showing weak or no inhibition even at the highest volume.

Substantial variation in antibacterial activity was observed among fungal isolates. Notably, *Fusarium* sp. GYP-36 exhibited the strongest inhibition against *E. coli* (31 mm at 20 μL) and *S. aureus* (27 mm at 20 μL), while maintaining moderate activity against *P. aeruginosa*. Other isolates, including *Colletotrichum* sp. GYP-28 and Botryosphaeriaceae sp. GYP-27, also showed relatively strong activity against *E. coli*. In contrast, only a limited number of isolates, such as *Gibellulopsis* sp. GYP-48 and *Talaromyces* sp. GYP-18, exhibited noticeable inhibition against *P. aeruginosa*.

Overall, the antibacterial activity of endophytic fungal extracts was strongly strain-dependent and varied considerably among target bacteria, with a consistent increase in activity at higher application volumes. This variability was further examined using multivariate analysis. PERMANOVA analysis confirmed that fungal taxonomic identity had a statistically significant effect on antibacterial activity profiles (Pseudo-F = 2.3231, *P* = 0.044), while host species and tissue origin did not show significant effects.

### 3.5 Minimum inhibitory concentration (MIC) of representative active isolates

Based on the disk diffusion assay results, the top endophytic fungal isolates with the strongest antibacterial activity were selected for MIC determination. *Fusarium* sp. GYP-36 exhibited MIC values of 64 μg mL^–1^ against both *E. coli* and *S. aureus*, further confirming its potent broad-spectrum antibacterial activity. In contrast, the same isolate showed only weak activity against *P. aeruginosa* (MIC > 128 μg mL^–1^), consistent with the disk diffusion results.

The positive control antibiotics showed expected MIC values. Against *E. coli*, penicillin sodium had an MIC of 64 μg mL^–1^, vancomycin hydrochloride > 128 μg mL^–1^, and gentamicin sulfate 4 μg mL^–1^. Against *S. aureus*, penicillin sodium had an MIC of 16 μg mL^–1^, vancomycin hydrochloride 2 μg mL^–1^, and gentamicin sulfate 2 μg mL^–1^. Against *P. aeruginosa*, penicillin sodium had an MIC of 64 μg mL^–1^, vancomycin hydrochloride > 128 μg mL^–1^, and gentamicin sulfate 4 μg mL^–1^. These results are consistent with standard susceptibility profiles and validate the assay conditions.

### 3.6 Principal component analysis of antibacterial activity profiles

Principal component analysis (PCA) was performed to explore variation in antibacterial activity profiles among endophytic fungal isolates.

For host species, the first two principal components (PC1 and PC2) explained 76.14% of the total variance (PC1: 47.49%; PC2: 28.65%). Although a partial separation between isolates from *G. pentaphyllum* and *G. longipes* was observed (Supplementary Figure 3A), PERMANOVA analysis indicated that the effect of host species was not statistically significant (Pseudo-F = 1.1589, *P* = 0.307).

For tissue origin, PCA showed substantial overlap among samples (Supplementary Figure 3B), which was consistent with PERMANOVA results indicating no significant effect (Pseudo-F = 0.3801, *P* = 0.879).

**FIGURE 3:**
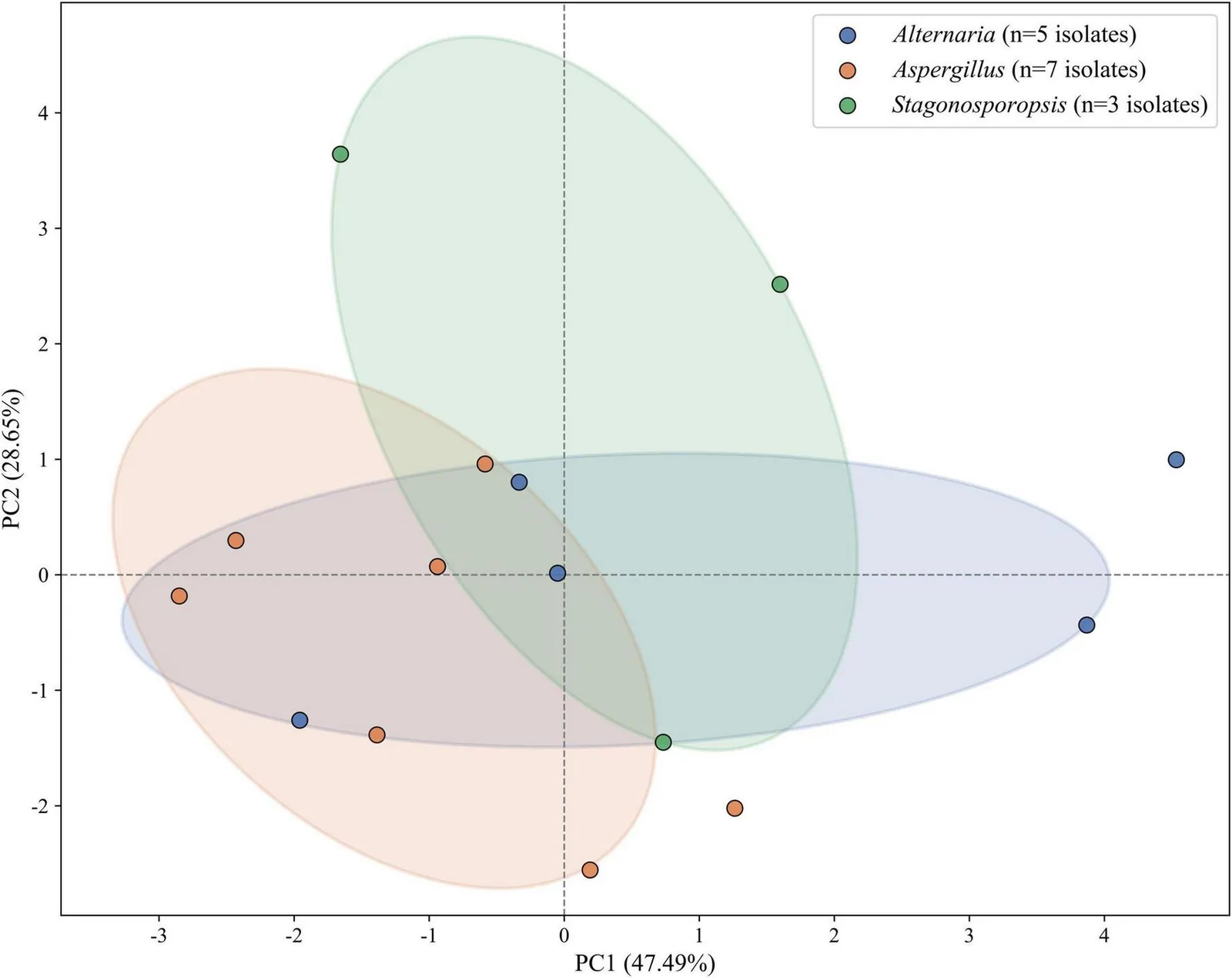
Principal component analysis (PCA) of antibacterial activity profiles of endophytic fungal isolates grouped by fungal taxa. PCA was performed based on standardized inhibition zone diameters against *Escherichia coli*, *Staphylococcus aureus*, and *Pseudomonas aeruginosa*. Each point represents an individual fungal isolate. Ellipses represent 95% confidence intervals and are shown for visualization purposes only. PERMANOVA analysis indicated that fungal taxonomic identity had a statistically significant effect on antibacterial activity profiles (Pseudo-F = 2.3231, *P* = 0.044).

In contrast, fungal taxa exhibited a clearer separation pattern in the PCA ordination space (Figure 3). PERMANOVA confirmed that fungal taxa had a statistically significant effect on antibacterial activity profiles (Pseudo-F = 2.3231, *P* = 0.044).

Fisher separability analysis showed a consistent trend, with fungal taxa exhibiting the highest separability (0.4694), compared with host species (0.0535), tissue origin (0.0223), and aboveground versus belowground grouping (0.0145).

Taken together, these results indicate that fungal taxa is the primary determinant of antibacterial activity variation, whereas host species and tissue origin do not have statistically significant effects. These findings should be interpreted with consideration that the observed separation for host and tissue factors was not statistically supported.

### 3.7 Characterization of antibacterial activity patterns in endophytic fungal isolates using heatmap analysis

The heatmap based on raw inhibition zone diameters (Supplementary Figure 4) showed a general tendency for fungal isolates to group into clusters with relatively high and low antibacterial activity levels. Isolates in the high-activity group exhibited consistently stronger inhibition across multiple bacterial species and application volumes, suggesting a broad-spectrum activity profile, whereas those in the low-activity group showed weak or negligible inhibition under most tested conditions.

Comparison across bacterial species and application volumes revealed heterogeneous and asymmetric patterns of bacterial susceptibility. *Staphylococcus aureus* was generally more susceptible to fungal extracts, with a relatively large proportion of isolates showing inhibitory activity. In contrast, *E. coli* exhibited intermediate susceptibility, with stronger inhibition observed in a subset of isolates and becoming more pronounced at higher application volumes. *Pseudomonas aeruginosa* displayed lower overall susceptibility and a more selective response pattern, with strong inhibition limited to a small number of isolates.

**FIGURE 4:**
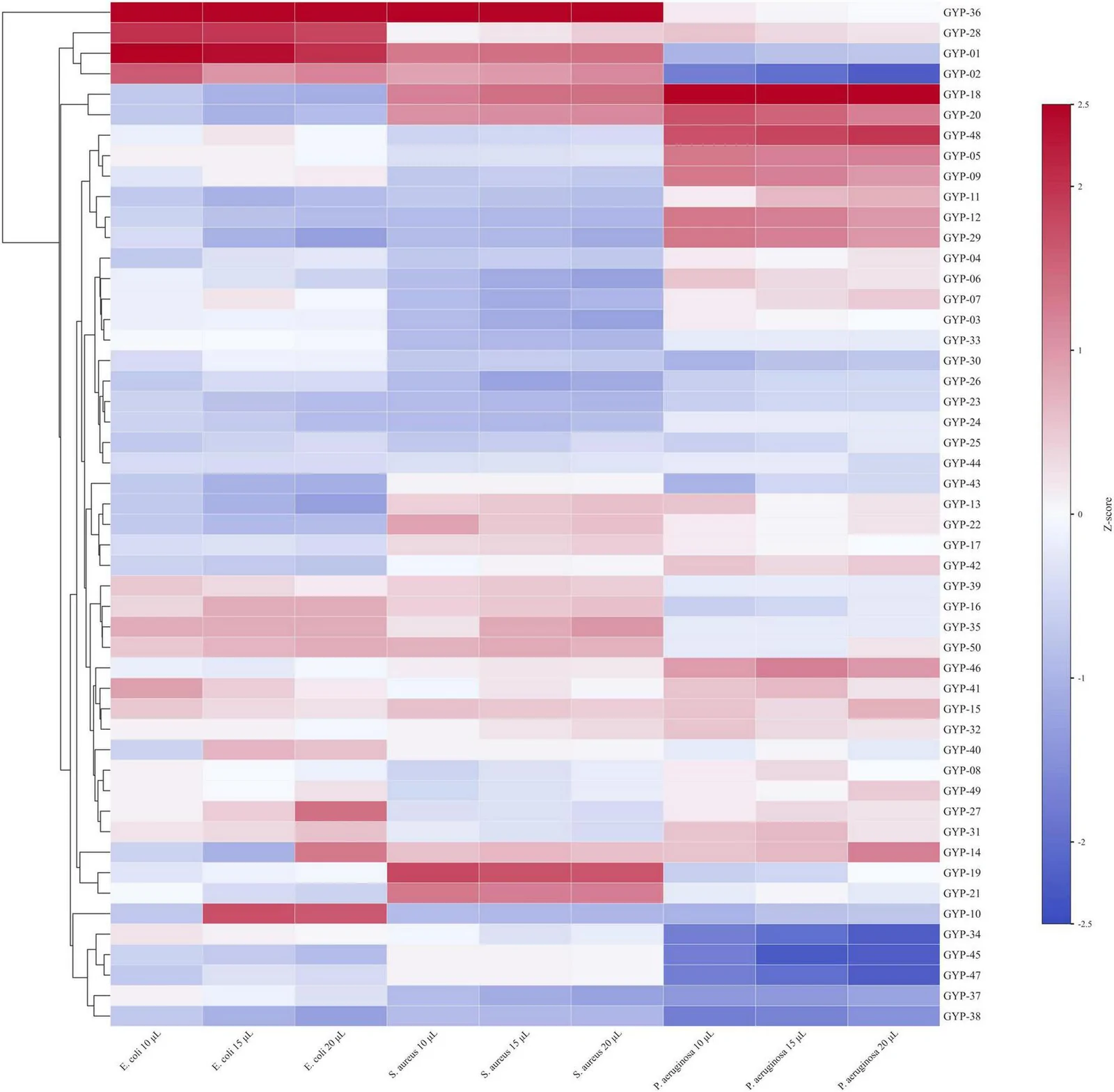
Hierarchical clustering heatmap of antibacterial activity profiles of endophytic fungal isolates. The heatmap is based on inhibition zone diameters (mm) against *Escherichia coli*, *Staphylococcus aureus*, and *Pseudomonas aeruginosa* at three application volumes (10, 15, and 20 μL). Data were standardized using Z-score normalization (column-wise). Hierarchical clustering was performed using Euclidean distance and Ward’s linkage method. The color scale represents relative antibacterial activity levels, with red indicating above-average activity, blue indicating below-average activity, and white representing the mean activity level. Rows represent individual fungal isolates, and columns represent bacterial species and application volumes. Clustering is shown for visualization of overall patterns and was not subjected to formal statistical testing.

The Z-score–normalized heatmap (Figure 4) further highlighted the relative distribution of activity levels among isolates. Highly active isolates tended to cluster in the upper region of the heatmap, exhibiting consistently positive Z-scores across multiple conditions, whereas low-activity isolates were more frequently observed in the lower region, showing predominantly negative Z-scores. For example, *Fusarium* sp. GYP-36 consistently exhibited high activity, whereas *Plectosphaerella* sp. GYP-38 showed relatively low activity across conditions.

Notably, some isolates exhibited condition-dependent variation in activity, showing strong inhibition under specific bacterial or application volume conditions but reduced activity elsewhere, indicating substantial strain-level heterogeneity. Representative examples include *Fusarium* sp. GYP-14, which exhibited stronger inhibition against *E. coli* at higher application volumes, and *Stagonosporopsis* sp. GYP-19, which showed pronounced activity against *S. aureus* but weaker responses against other bacterial species.

In addition, an overall increase in inhibition intensity from 10 to 20 μL was observed for some active isolates, although this trend was not consistent across all strains or bacterial targets.

Overall, the heatmap provides a descriptive overview of antibacterial activity patterns, highlighting variability among fungal isolates, asymmetric susceptibility across bacterial species, volume-dependent effects, and pronounced strain-level heterogeneity.

### 3.8 Ranking of top 10 isolates based on antibacterial activity

**FIGURE 5:**
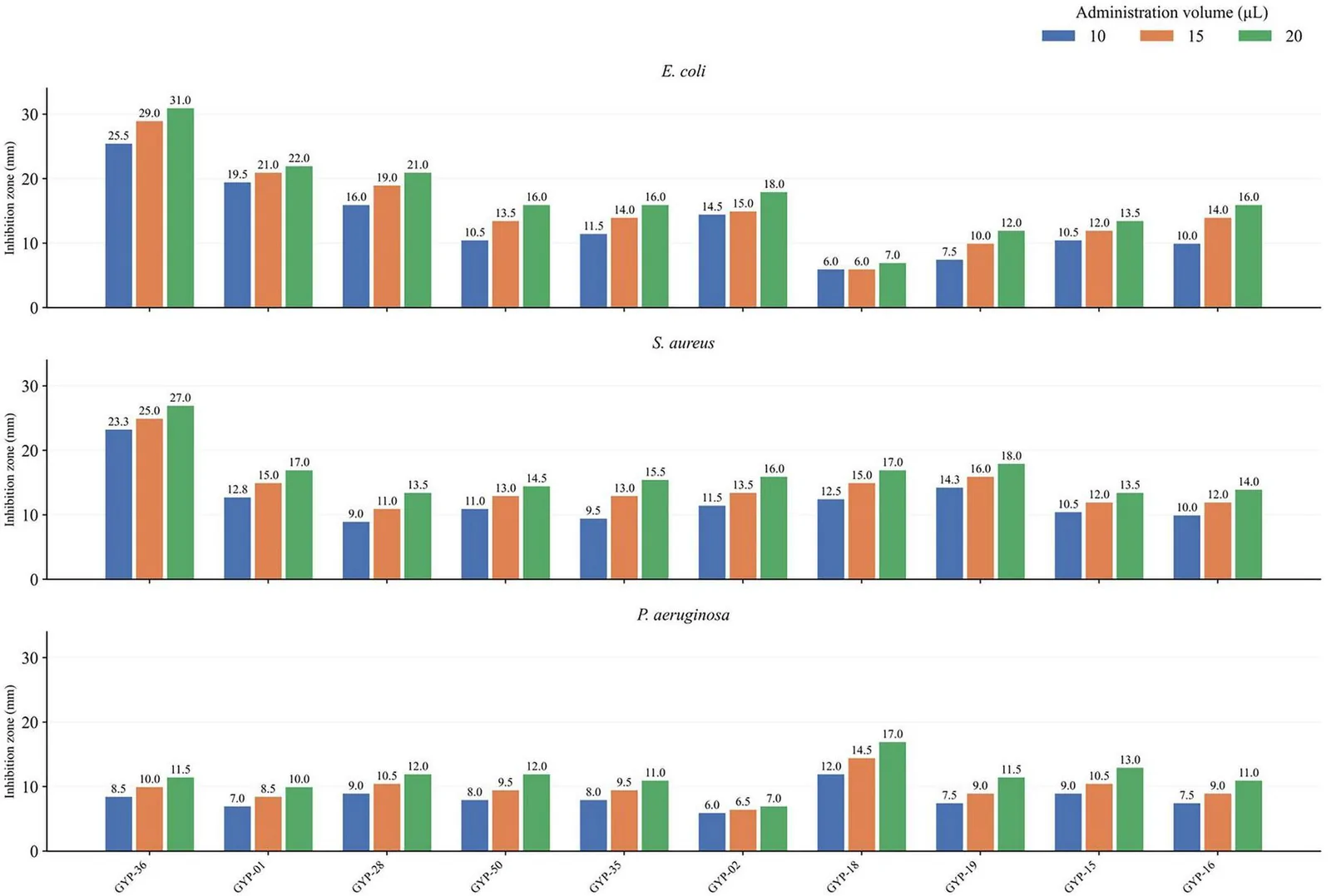
Top 10 ranking of endophytic fungal isolates based on their average antibacterial activity. The ranking score for each isolate was calculated as the average inhibition zone diameter (mm) across all bacterial species (*E. coli*, *S. aureus*, *P. aeruginosa*) and application volumes (10 μL, 15 μL, and 20 μL). Isolates are ranked from highest to lowest activity based on their overall performance. Bar charts show the inhibition zone diameters (mm) for each fungal extract against *E. coli*, *S. aureus*, and *P. aeruginosa*. Data are presented for three application volumes: 10 μL (blue), 15 μL (orange), and 20 μL (green).

Based on the activity patterns revealed by the heatmap analysis, endophytic fungal isolates were ranked according to the average inhibition zone diameter (mm) calculated from the inhibition zones measured against *E. coli*, *S. aureus*, and *P. aeruginosa* at three different application volumes (10 μL, 15 μL, and 20 μL). The top 10 isolates are presented in Figure 5. Among them, *Fusarium* sp. GYP-36 exhibited the strongest activity, producing substantially larger inhibition zones against *E. coli* and *S. aureus* across all tested application volumes.

The ranked isolates displayed distinct activity profiles, with noticeable variation across bacterial species and application volumes. Some isolates showed broad-spectrum activity, maintaining relatively high inhibition against all three bacterial species, whereas others exhibited more variable or moderately selective responses. For example, *Fusarium* sp. GYP-36 and *Fusarium* sp. GYP-35 can be regarded as broad-spectrum isolates, showing comparable inhibitory effects across all tested bacteria. In contrast, *Talaromyces* sp. GYP-18, *Stagonosporopsis* sp. GYP-19, and *Colletotrichum* sp. GYP-28 exhibited preferential activity toward *P. aeruginosa*, *S. aureus*, and *E. coli*, respectively (Figure 5).

Consistent with the patterns observed in previous analyses, *S. aureus* was the most susceptible species, with most isolates showing moderate to strong inhibition across application volumes. *Escherichia coli* exhibited intermediate sensitivity, whereas *P. aeruginosa* remained comparatively less susceptible overall, with pronounced inhibition observed only in a limited number of isolates.

A clear dose-dependent trend was observed across all isolates, with inhibition generally increasing with application volume, although the magnitude of this effect varied among isolates and bacterial species. For example, *Fusarium* sp. GYP-36 showed a clear and consistent increase across all three bacterial species, whereas *Alternaria* sp. GYP-02 exhibited a more gradual response against *P. aeruginosa*.

## 4 Discussion

*Gynostemma* possesses rich phytochemical constituents and diverse pharmacological activities (Su et al., 2021). Its associated endophytic fungi exhibit considerable functional potential, including enzymatic biotransformation and production of polysaccharides and bioactive metabolites with antioxidant, antibacterial, and anti-inflammatory activities (Fan, 2013; Ma et al., 2015; Zhang et al., 2017, 2023; Wang et al., 2019, 2021). However, comparative studies on endophytic fungal composition among different *Gynostemma* species remain limited.

In the present study, we integrated host chemical profiles, endophytic fungal community composition, and antibacterial activities to compare two closely related *Gynostemma* species. Our results revealed distinct differences between *G. pentaphyllum* and *G. longipes* in both metabolite profiles and endophytic fungal communities. Specifically, *G. pentaphyllum*, which exhibited higher saponin content, harbored greater fungal diversity than *G. longipes*, whereas *G. longipes* showed relatively higher flavonoid content and lower fungal diversity. These findings suggest a potential association between host metabolite profiles and variations in endophytic fungal community composition across the two *Gynostemma* species. However, whether specific metabolites are involved in generating these differences and the underlying mechanisms remain to be elucidated. Previous studies on medicinal plants, including *Panax notoginseng* and *Curcuma longa*, have suggested that microbial community composition is associated with host chemical characteristics. Plant metabolites (particularly root exudates) may alter the plant microenvironment. This difference in chemical environment can influence the enrichment or depletion of specific microorganisms (Hardoim et al., 2015; Broeckling et al., 2008). Although the specific relationships between individual metabolites and fungal community composition require further investigation, our comparative analysis of two closely related *Gynostemma* species with distinct chemical profiles and endophytic fungal communities expands our understanding of *Gynostemma*-associated endophytic fungi and provides new insights into the potential relationship between host chemical traits and fungal community variation.

The composition of the endophytic fungal communities associated with *Gynostemma* differed between the two host species, with *Alternaria* and *Aspergillus* representing dominant genera in *G. pentaphyllum* and *G. longipes*, respectively. The predominance of *Alternaria* in *G. pentaphyllum* is consistent with previous studies on endophytic fungi associated with *Gynostemma*, in which *Alternaria* was also reported as one of the dominant fungal genera (Fan, 2013; Jeewon et al., 2013). These findings suggest that certain dominant fungal taxa, such as *Alternaria*, may represent frequently detected members of the endophytic fungal communities associated with *Gynostemma* hosts. However, the mechanisms underlying their distribution patterns remain unclear. Although *Aspergillus* showed a relatively higher abundance in *G. longipes* in the present study, the difference in dominant fungal taxa between the two closely related *Gynostemma* species suggests that even phylogenetically related hosts may harbor distinct endophytic fungal communities. Whether these differences are associated with host biological characteristics or other factors requires further investigation (Jeewon et al., 2004, 2017).

In terms of antimicrobial activity, *Fusarium* sp. GYP-36 exhibited significant broad-spectrum antibacterial effects against both Gram-positive and Gram-negative bacteria, with inhibition zones of up to 31 mm against *E. coli* and 27 mm against *S. aureus*. The MIC assay further confirmed its antibacterial potential, with MIC values of 64 μg mL^–1^ against both tested bacterial strains. Similar antimicrobial activities have been reported in other *Fusarium* species isolated from medicinal plants, which produce various bioactive metabolites, including fusaric acid, beauvericin, and enniatins (Jeewon et al., 2024; Moglad et al., 2023; Summerell, 2019). These findings further highlight the potential of *Fusarium* species as valuable resources for the discovery of antimicrobial compounds.

Despite these findings, several limitations should be acknowledged. First, although correlations between host metabolite profiles and endophytic fungal community variation were observed, the causal relationships remain unclear and require further experimental validation. Second, although *Fusarium* sp. GYP-36 exhibited strong antibacterial activity, its active metabolites and mechanisms remain to be characterized. Furthermore, this study focused on two *Gynostemma* species from the Pingli region of Shaanxi Province, and broader investigations are needed to further evaluate the generality of these findings.

Overall, this study provides valuable insights into the interactions between host plants and their endophytes, highlighting the potential of *Gynostemma* species as sources of bioactive endophytic fungi for antimicrobial discovery. The potent antibacterial activity exhibited by *Fusarium* sp. GYP-36 highlights its potential as a promising source for the discovery of natural antimicrobial agents.

## 5 Conclusion

This study revealed significant differences in endophytic fungal diversity and community composition between *G. pentaphyllum* and *G. longipes*. Antibacterial activity was primarily associated with fungal taxonomic identity rather than host species or tissue origin. Notably, *Fusarium* sp. GYP-36 exhibited strong broad-spectrum antibacterial activity, highlighting its potential as a promising candidate for antimicrobial development. These findings provide new insights into host–endophyte interactions and underscore the potential of medicinal plants as valuable reservoirs of bioactive microorganisms.

## Data Availability

The datasets presented in this study can be found in online repositories. The names of the repository/repositories and accession number(s) can be found below: https://www.ncbi.nlm.nih.gov/, PZ285361-PZ285409.
