# Supplementary material for: Diversity, community composition, and antibacterial activity of endophytic fungi isolated from two Gynostemma species
Source: Front Microbiol. 2026 Aug 24;17:1867726. doi: 10.3389/fmicb.2026.1867726 (PMC13547514; doi:10.3389/fmicb.2026.1867726)
Supplement: Supplementary file 1 [file Table_1.docx]

Supplementary Material

# 1 Supplementary Note

# Note S1. Calculation of ecological diversity indices

$$\text{CR}\text{=}\frac{\text{Number}\text{ }\text{of}\text{ }\text{fragments}\text{ }\text{colonized}}{\text{Number}\text{ }\text{of}\text{ }\text{fragments}\text{ }\text{incubated}}$$

$$\text{H}\text{'=−}\sum_{\text{i}\text{=1}}^{\text{S}} \text{p}_{\text{i}}\ln\text{p}_{\text{i}}$$

$$\text{J}\text{=}\frac{\text{H}\text{'}}{\ln\text{(}\text{S}\text{ )}}$$

$$\text{D}\text{=1−}\sum_{\text{i}\text{=1}}^{\text{S}} \text{p}_{\text{i}}^{\text{2}}$$

$$\text{IR}\text{=}\frac{\text{Total}\text{ }\text{Number}\text{ }\text{of}\text{ }\text{isolates}}{\text{Number}\text{ }\text{of}\text{ }\text{fragments}\text{ }\text{incubated}}$$

where pi is the proportion of individuals belonging to the i-th species in the sample $\left( \text{p}_{\text{i}}\text{=}\frac{\text{n}_{\text{i}}}{\text{N}} \right)$, with ni being the number of isolates of the i-th species and N being the total number of isolates in the sample.

# 2 Supplementary Figures and Tables

**Table S1.** Morphological characteristics of *Gynostemma pentaphyllum* and *Gynostemma longipes*

| Species | Fruit Stalk Length | Leaf Characteristics | Fruits Characteristics |
| --- | --- | --- | --- |
| *G. pentaphyllum* | <5 mm | Mostly 5 leaflets;  petiole 1–4 cm;  margins shallowly serrated | Diameter 5–6 mm; smooth surface |
| *G. longipes* | 15–20 mm | 3, 5, or 7 leaflets;  petiole 4–10 cm; margins distinctly serrated | Diameter 6–8 mm; textured surface |

**Table S2.** Colorimetric determination conditions for major bioactive components in *Gynostemma* species

| **Major Component** | **Method** | **Detection Wavelength (nm)** | **Reference Standard** |
| --- | --- | --- | --- |
| Saponins | Vanillin–perchloric acid | 550 | Ginsenoside Rb1 |
| Polysaccharides | Anthrone–sulfuric acid | 625 | D-Glucose |
| Flavonoids | Aluminum nitrate colorimetric system | 510 | Rutin |

**Figure S1.** Tissues of two *Gynostemma* species used for the isolation of endophytic fungi. (a) Leaves, (b) Stems, (c) Roots, (d) Fibrous roots, (e) Shoot tips.

**Table S3.** Tissue-specific distribution of major bioactive components in two *Gynostemma* species (%)

| Species | Tissue | Saponins | Polysaccharides | Flavonoids |
| --- | --- | --- | --- | --- |
| *G. pentaphyllum* | Leaf | 16.5 | 6.1 | 3.9 |
|  | Stem | 13.0 | 5.5 | 2.3 |
|  | Main root | 5.2 | 4.8 | 1.8 |
|  | Fibrous root | 4.0 | 7.8 | 1.2 |
|  | Shoot tip | 3.3 | 8.6 | 1.0 |
|  | Total | 12.0^a^ | 6.89 | 2.35^a^ |
| *G. longipes* | Leaf | 7.8 | 6.3 | 4.5 |
|  | Stem | 6.2 | 5.7 | 3.7 |
|  | Main root | 2.9 | 4.6 | 3.2 |
|  | Fibrous root | 2.3 | 7.2 | 1.1 |
|  | Shoot tip | 1.8 | 8.0 | 0.9 |
|  | Total | 4.50^b^ | 6.70 | 3.82^b^ |

Note: Values are expressed as percentage (%) and represent the mean of three replicates. Superscript letters represent statistically significant differences at P < 0.05 (for total saponins and total flavonoids).

**Table S4.** Closest GenBank matches and isolation information of endophytic fungal isolates from two *Gynostemma* species

| **Fungal isolate** | **Closest GenBank Matches**  **(ITS database)** | **Similarity**  **(%)** | **Closest GenBank Matches**  **(core_nt database)** | **Similarity**  **(%)** | **Tissue** | **Host Plant** |
| --- | --- | --- | --- | --- | --- | --- |
| *Alternaria* sp. GYP-01 | *Alternaria* spp.  (*A. doliconidium*, *A. destruens*) | 100.00 | *Alternaria* spp.  (*A. [alternata](https://www.ncbi.nlm.nih.gov/Taxonomy/Browser/wwwtax.cgi?id=5599" \o "Taxonomy for Alternaria alternata" \t "https://blast.ncbi.nlm.nih.gov/lnk<@rid@>)*, *A. pomicola*, etc.) | 100.00 | main roots shoot apices fibrous roots | *G. pentaphyllum* |
| *Alternaria* sp. GYP-02 | *Alternaria [destruens](https://www.ncbi.nlm.nih.gov/Taxonomy/Browser/wwwtax.cgi?id=230003" \o "Taxonomy for Alternaria destruens" \t "https://blast.ncbi.nlm.nih.gov/lnk<@rid@>)* | 99.62 | *Alternaria* spp.  (*A. [alternata](https://www.ncbi.nlm.nih.gov/Taxonomy/Browser/wwwtax.cgi?id=5599" \o "Taxonomy for Alternaria alternata" \t "https://blast.ncbi.nlm.nih.gov/lnk<@rid@>)*, *A. tenuissima*) | 99.81 | leaves main roots fibrous roots | *G. pentaphyllum* |
| *Alternaria* sp. GYP-03 | *Alternaria* spp.  (*A. alstroemeriae*, *A. prunicola*, etc.) | 99.80 | *Alternaria* spp.  (*A. [alternata](https://www.ncbi.nlm.nih.gov/Taxonomy/Browser/wwwtax.cgi?id=5599" \o "Taxonomy for Alternaria alternata" \t "https://blast.ncbi.nlm.nih.gov/lnk<@rid@>)*, *A. [arborescens](https://www.ncbi.nlm.nih.gov/Taxonomy/Browser/wwwtax.cgi?command=show&mode=node&id=156630&lvl=)*, etc.) | 100.00 | leaves | *G. pentaphyllum* |
| *Colletotrichum* sp. GYP-04 | *Colletotrichum* spp.  (*C. utrechtens*e, *C. higginsianum*, etc.) | 99.60 | *Colletotrichum* spp.  (*C. neorubicola*, *C. higginsianum*, etc.) | 99.60 | fibrous roots | *G. pentaphyllum* |
| *Colletotrichum* sp. GYP-05 | *Colletotrichum* spp.  (*C. truncatum*, *C. aciculare*) | 100.00 | *Colletotrichum* spp.  (*C. truncatum*, *C. jasminigenum*, etc) | 100.00 | stems fibrous roots | *G. pentaphyllum* |
| *Colletotrichum* sp. GYP-06 | *Colletotrichum jasminigenum* | 99.26 | *Colletotrichum* spp.  (*C. truncatum*, *C. jasminigenum*, etc) | 99.45 | stems | *G. pentaphyllum* |
| *Colletotrichum* sp. GYP-07 | *Colletotrichum* spp.  (*C. merremiae*, *C. [brevisporum](https://www.ncbi.nlm.nih.gov/Taxonomy/Browser/wwwtax.cgi?id=1125787" \o "Taxonomy for Colletotrichum brevisporum" \t "https://blast.ncbi.nlm.nih.gov/lnk<@rid@>)*) | 99.61 | *Colletotrichum liaoningense* | 99.80 | stems | *G. pentaphyllum* |
| *Colletotrichum* sp. GYP-08 | *Colletotrichum* spp.  (*[C. ainanense](https://www.ncbi.nlm.nih.gov/Taxonomy/Browser/wwwtax.cgi?id=2707334" \o "Taxonomy for Colletotrichum tainanense" \t "https://blast.ncbi.nlm.nih.gov/lnk<@rid@>)*, *C. yulongense*, etc.) | 100.00 | *Colletotrichum* spp.  (*C. siamense*, *C. asianum*, etc) | 100.00 | leaves | *G. pentaphyllum* |
| *Aspergillus* sp. GYP-09 | *Aspergillus sydowii* | 100.00 | *Aspergillus* spp.  (*A. sydowii*, *[A. versicolor](https://www.ncbi.nlm.nih.gov/Taxonomy/Browser/wwwtax.cgi?id=1341132" \o "Taxonomy for Aspergillus welwitschiae" \t "https://blast.ncbi.nlm.nih.gov/lnk<@rid@>)*, etc.) | 100.00 | fibrous roots | *G. pentaphyllum* |
| *Aspergillus* sp. GYP-10 | *Aspergillus* spp.  (*A. foetidus*, *[A. welwitschiae](https://www.ncbi.nlm.nih.gov/Taxonomy/Browser/wwwtax.cgi?id=1341132" \o "Taxonomy for Aspergillus welwitschiae" \t "https://blast.ncbi.nlm.nih.gov/lnk<@rid@>)*, etc.) | 93.28 | *Aspergillus* spp.  (*A. niger*, *[A. welwitschiae](https://www.ncbi.nlm.nih.gov/Taxonomy/Browser/wwwtax.cgi?id=1341132" \o "Taxonomy for Aspergillus welwitschiae" \t "https://blast.ncbi.nlm.nih.gov/lnk<@rid@>)*, etc.) | 93.28 | stems | *G. pentaphyllum* |
| *Aspergillus* sp. GYP-11 | *Aspergillus [sydowii](https://www.ncbi.nlm.nih.gov/Taxonomy/Browser/wwwtax.cgi?id=75750" \o "Taxonomy for Aspergillus sydowii" \t "https://blast.ncbi.nlm.nih.gov/lnk<@rid@>)* | 99.61 | *Aspergillus* spp.  (*A. sydowii*, *[A. versicolor](https://www.ncbi.nlm.nih.gov/Taxonomy/Browser/wwwtax.cgi?id=1341132" \o "Taxonomy for Aspergillus welwitschiae" \t "https://blast.ncbi.nlm.nih.gov/lnk<@rid@>)*, etc.) | 99.62 | stems main roots shoot apices fibrous roots | *G. pentaphyllum* |
| *Fusarium* sp. GYP-12 | *Fusarium longifundum* | 99.78 | *Fusarium* spp.  (*F. longifundum*, *[F.](https://www.ncbi.nlm.nih.gov/Taxonomy/Browser/wwwtax.cgi?id=1341132" \o "Taxonomy for Aspergillus welwitschiae" \t "https://blast.ncbi.nlm.nih.gov/lnk<@rid@>) [graminearum](https://www.ncbi.nlm.nih.gov/Taxonomy/Browser/wwwtax.cgi?command=show&mode=node&id=5518&lvl=)*, etc.) | 100.00 | fibrous roots | *G. pentaphyllum* |
| *Fusarium* sp. GYP-13 | *Fusarium solani* | 100.00 | *Fusarium* spp.  (*F. solani*, *[F.](https://www.ncbi.nlm.nih.gov/Taxonomy/Browser/wwwtax.cgi?id=1341132" \o "Taxonomy for Aspergillus welwitschiae" \t "https://blast.ncbi.nlm.nih.gov/lnk<@rid@>) [oxysporum](https://www.ncbi.nlm.nih.gov/Taxonomy/Browser/wwwtax.cgi?command=show&mode=node&id=5507&lvl=)*, etc.) | 100.00 | shoot apices | *G. pentaphyllum* |
| *Fusarium* sp. GYP-14 | *Fusarium [hordeum](https://www.ncbi.nlm.nih.gov/Taxonomy/Browser/wwwtax.cgi?id=2773086" \o "Taxonomy for Fusarium hordeum" \t "https://blast.ncbi.nlm.nih.gov/lnk<@rid@>)* | 99.55 | *Fusarium* spp.  (*F. foetens*, *[F.](https://www.ncbi.nlm.nih.gov/Taxonomy/Browser/wwwtax.cgi?id=1341132" \o "Taxonomy for Aspergillus welwitschiae" \t "https://blast.ncbi.nlm.nih.gov/lnk<@rid@>) [oxysporum](https://www.ncbi.nlm.nih.gov/Taxonomy/Browser/wwwtax.cgi?command=show&mode=node&id=5507&lvl=)*, etc.) | 100.00 | shoot apices | *G. pentaphyllum* |
| *Penicillium* sp. GYP-15 | *Penicillium rubens* | 100.00 | *Penicillium* spp.  (*[P.](https://www.ncbi.nlm.nih.gov/Taxonomy/Browser/wwwtax.cgi?id=1341132" \o "Taxonomy for Aspergillus welwitschiae" \t "https://blast.ncbi.nlm.nih.gov/lnk<@rid@>) rubens*, *[P.](https://www.ncbi.nlm.nih.gov/Taxonomy/Browser/wwwtax.cgi?id=1341132" \o "Taxonomy for Aspergillus welwitschiae" \t "https://blast.ncbi.nlm.nih.gov/lnk<@rid@>) goetzii*, etc.) | 100.00 | stems leaves main roots shoot apices fibrous roots | *G. pentaphyllum* |
| *Penicillium* sp. GYP-16 | *Penicillium citrinum* | 93.60 | *Penicillium citrinum* | 93.62 | shoot apices | *G. pentaphyllum* |
| Trichocomaceae sp. GYP-17 | *Talaromyces californicus* | 99.80 | Several Trichocomaceae taxa (*Talaromyces*, *Penicillium*, etc.) | 100.00 | shoot apices | *G. pentaphyllum* |
| *Talaromyces* sp. GYP-18 | *Talaromyces [assiutensis](https://www.ncbi.nlm.nih.gov/Taxonomy/Browser/wwwtax.cgi?id=91640" \o "Taxonomy for Talaromyces assiutensis" \t "https://blast.ncbi.nlm.nih.gov/lnk<@rid@>)* | 99.41 | *Talaromyces trachyspermus* | 100.00 | stems | *G. pentaphyllum* |
| *Stagonosporopsis* sp. GYP-19 | *Stagonosporopsis* spp.  (*S. dorenboschii*, *S. loticola*, *S. lupini*) | 98.77 | *Stagonosporopsis* sp. | 100.00 | stems leaves fibrous roots | *G. pentaphyllum* |
| *Stagonosporopsis* sp. GYP-20 | *Stagonosporopsis [ailanthicola](https://www.ncbi.nlm.nih.gov/Taxonomy/Browser/wwwtax.cgi?id=2043533" \o "Taxonomy for Stagonosporopsis ailanthicola" \t "https://blast.ncbi.nlm.nih.gov/lnk<@rid@>)* | 98.90 | *Stagonosporopsis* sp. | 100.00 | main roots fibrous roots | *G. pentaphyllum* |
| *Trichoderma* sp. GYP-21 | *Trichoderma* spp.  (*T. evansii*, *T. [hamatum](https://www.ncbi.nlm.nih.gov/Taxonomy/Browser/wwwtax.cgi?id=49224" \o "Taxonomy for Trichoderma hamatum" \t "https://blast.ncbi.nlm.nih.gov/lnk<@rid@>)*) | 99.81 | *Trichoderma* spp.  (*T. asperellum*, *T. [hamatum](https://www.ncbi.nlm.nih.gov/Taxonomy/Browser/wwwtax.cgi?id=49224" \o "Taxonomy for Trichoderma hamatum" \t "https://blast.ncbi.nlm.nih.gov/lnk<@rid@>)*, etc) | 100.00 | shoot apices | *G. pentaphyllum* |
| *Neofusicoccum* sp. GYP-22 | *Neofusicoccum parvum* | 99.60 | *Neofusicoccum* spp.  (*N. parvum*, *N. yunnanense*, etc) | 99.80 | fibrous roots | *G. pentaphyllum* |
| Phaeosphaeriaceae sp. GYP-23 | *Acericola italica* | 90.46 | Several Phaeosphaeriaceae taxa  (*Acericola*, *Phaeosphaeria*, etc.) | 100.00 | leaves | *G. pentaphyllum* |
| *Paramyrothecium* sp. GYP-24 | *Paramyrothecium viridisporum* | 99.61 | *Paramyrothecium* spp.  (*P. sinense*, *P. viridisporum*, etc.) | 100.00 | stems | *G. pentaphyllum* |
| *Ceratobasidium* sp. GYP-25 | *Ceratobasidium [occidentalis](https://www.ncbi.nlm.nih.gov/Taxonomy/Browser/wwwtax.cgi?id=1938109" \o "Taxonomy for Ceriporia occidentalis" \t "https://blast.ncbi.nlm.nih.gov/lnk<@rid@>)* | 99.40 | *Ceratobasidium* sp. | 99.49 | stems shoot apices fibrous roots | *G. pentaphyllum* |
| *Plectosphaerella* sp. GYP-26 | *Plectosphaerella cucumerina* | 99.81 | *Plectosphaerella* spp.  (*P. cucumerina*, *P. niemeijerarum*, etc) | 100.00 | stems | *G. pentaphyllum* |
| Botryosphaeriaceae sp. GYP-27 | *Phyllosticta* spp.  (*P. Fallopiae*, *P. [capitalensis](https://www.ncbi.nlm.nih.gov/Taxonomy/Browser/wwwtax.cgi?id=121624" \o "Taxonomy for Phyllosticta capitalensis" \t "https://blast.ncbi.nlm.nih.gov/lnk<@rid@>)*) | 100.00 | Several Botryosphaeriaceae taxa  (*Phyllosticta*, *Botryosphaeria*, etc.) | 100.00 | main roots leaves | *G. pentaphyllum* |
| *Colletotrichum* sp. GYP-28 | *Colletotrichum* spp.  (*C.[doitungense](https://www.ncbi.nlm.nih.gov/Taxonomy/Browser/wwwtax.cgi?id=2152657" \o "Taxonomy for Colletotrichum doitungense" \t "https://blast.ncbi.nlm.nih.gov/lnk<@rid@>)*, *C.[watphraense](https://www.ncbi.nlm.nih.gov/Taxonomy/Browser/wwwtax.cgi?id=2152659" \o "Taxonomy for Colletotrichum watphraense" \t "https://blast.ncbi.nlm.nih.gov/lnk<@rid@>)*) | 99.61 | *Colletotrichum* spp.  (*C.boninense*, *C.[gloeosporioides](https://www.ncbi.nlm.nih.gov/Taxonomy/Browser/wwwtax.cgi?command=show&mode=node&id=474922&lvl=)*, etc) | 99.64 | leaves | *G. longipes* |
| *Colletotrichum* sp. GYP-29 | *Colletotrichum* spp.  (*C. yulongense*, *[C. cobbittiense](https://www.ncbi.nlm.nih.gov/Taxonomy/Browser/wwwtax.cgi?id=2510598" \o "Taxonomy for Colletotrichum cobbittiense" \t "https://blast.ncbi.nlm.nih.gov/lnk<@rid@>)*, etc.) | 100.00 | *Colletotrichum* spp.  (*C. siamense*, *C. asianum*, etc) | 100.00 | main roots | *G. longipes* |
| *Colletotrichum* sp. GYP-30 | *Colletotrichum* spp.  (*C. [makassarense](https://www.ncbi.nlm.nih.gov/Taxonomy/Browser/wwwtax.cgi?id=2707332" \o "Taxonomy for Colletotrichum makassarense" \t "https://blast.ncbi.nlm.nih.gov/lnk<@rid@>)*, *[C.](https://www.ncbi.nlm.nih.gov/Taxonomy/Browser/wwwtax.cgi?id=2510598" \o "Taxonomy for Colletotrichum cobbittiense" \t "https://blast.ncbi.nlm.nih.gov/lnk<@rid@>) [tainanense](https://www.ncbi.nlm.nih.gov/Taxonomy/Browser/wwwtax.cgi?id=2707334" \o "Taxonomy for Colletotrichum tainanense" \t "https://blast.ncbi.nlm.nih.gov/lnk<@rid@>)*, etc.) | 99.79 | *Colletotrichum* spp.  (*C. cangyuanense*, *C. henanense*, etc.) | 99.81 | leaves | *G. longipes* |
| *Aspergillus* sp. GYP-31 | *Aspergillus* spp.  (*A. costaricensis*, *[A. luchuensis](https://www.ncbi.nlm.nih.gov/Taxonomy/Browser/wwwtax.cgi?id=1069201" \o "Taxonomy for Aspergillus luchuensis" \t "https://blast.ncbi.nlm.nih.gov/lnk<@rid@>)*, etc.) | 100.00 | *Aspergillus* spp.  (*A. niger*, *[A.](https://www.ncbi.nlm.nih.gov/Taxonomy/Browser/wwwtax.cgi?id=1069201" \o "Taxonomy for Aspergillus luchuensis" \t "https://blast.ncbi.nlm.nih.gov/lnk<@rid@>) [tubingensis](https://www.ncbi.nlm.nih.gov/Taxonomy/Browser/wwwtax.cgi?command=show&mode=node&id=5068&lvl=)*, etc.) | 100.00 | fibrous roots | *G. longipes* |
| *Aspergillus* sp. GYP-32 | *Aspergillus [sydowii](https://www.ncbi.nlm.nih.gov/Taxonomy/Browser/wwwtax.cgi?id=75750" \o "Taxonomy for Aspergillus sydowii" \t "https://blast.ncbi.nlm.nih.gov/lnk<@rid@>)* | 99.61 | *Aspergillus* spp.  (*A. [sydowii](https://www.ncbi.nlm.nih.gov/Taxonomy/Browser/wwwtax.cgi?id=75750" \o "Taxonomy for Aspergillus sydowii" \t "https://blast.ncbi.nlm.nih.gov/lnk<@rid@>)*, *[A. amoenus](https://www.ncbi.nlm.nih.gov/Taxonomy/Browser/wwwtax.cgi?id=1069201" \o "Taxonomy for Aspergillus luchuensis" \t "https://blast.ncbi.nlm.nih.gov/lnk<@rid@>)*, etc.) | 99.62 | fibrous roots leaves main roots | *G. longipes* |
| *Aspergillus* sp. GYP-33 | *Aspergillus* spp.  (*A. foetidus*, *A. [welwitschiae](https://www.ncbi.nlm.nih.gov/Taxonomy/Browser/wwwtax.cgi?id=1341132" \o "Taxonomy for Aspergillus welwitschiae" \t "https://blast.ncbi.nlm.nih.gov/lnk<@rid@>)*, etc.) | 99.82 | *Aspergillus niger* | 100.00 | leaves | *G. longipes* |
| *Aspergillus* sp. GYP-34 | *Aspergillus* spp.  (*A. [dipodomyus](https://www.ncbi.nlm.nih.gov/Taxonomy/Browser/wwwtax.cgi?id=2713180" \o "Taxonomy for Aspergillus dipodomyus" \t "https://blast.ncbi.nlm.nih.gov/lnk<@rid@>)*, *[A. falconensi](https://www.ncbi.nlm.nih.gov/Taxonomy/Browser/wwwtax.cgi?id=1810910" \o "Taxonomy for Aspergillus falconensis" \t "https://blast.ncbi.nlm.nih.gov/lnk<@rid@>)*[s](https://www.ncbi.nlm.nih.gov/Taxonomy/Browser/wwwtax.cgi?id=1810910" \o "Taxonomy for Aspergillus falconensis" \t "https://blast.ncbi.nlm.nih.gov/lnk<@rid@>)) | 100.00 | *Aspergillus* spp.  (*A. [dipodomyus](https://www.ncbi.nlm.nih.gov/Taxonomy/Browser/wwwtax.cgi?id=2713180" \o "Taxonomy for Aspergillus dipodomyus" \t "https://blast.ncbi.nlm.nih.gov/lnk<@rid@>)*, *[A. dipodomyus](https://www.ncbi.nlm.nih.gov/Taxonomy/Browser/wwwtax.cgi?id=1810910" \o "Taxonomy for Aspergillus falconensis" \t "https://blast.ncbi.nlm.nih.gov/lnk<@rid@>)*[, ect](https://www.ncbi.nlm.nih.gov/Taxonomy/Browser/wwwtax.cgi?id=1810910" \o "Taxonomy for Aspergillus falconensis" \t "https://blast.ncbi.nlm.nih.gov/lnk<@rid@>) ) | 100.00 | fibrous roots stems main roots | *G. longipes* |
| *Fusarium* sp. GYP-35 | *Fusarium kyushuense* | 97.22 | *Fusarium kyushuense* | 100.00 | fibrous roots | *G. longipes* |
| *Fusarium* sp. GYP-36 | *Fusarium* spp.  (*F. foetens*, *F.oxysporum*, etc.) | 100.00 | *Fusarium* spp.  (*F. foetens*, *F.oxysporum*, etc.) | 100.00 | shoot apices stems | *G. longipes* |
| Nectriaceae sp. GYP-37 | *Fusarium solani* | 100.00 | Several Nectriaceae taxa  (*Fusarium*, *Neocosmospora*) | 100.00 | shoot apices stems | *G. longipes* |
| *Plectosphaerella* sp. GYP-38 | *Plectosphaerella [vigrensis](https://www.ncbi.nlm.nih.gov/Taxonomy/Browser/wwwtax.cgi?id=3074732" \o "Taxonomy for Plectosphaerella vigrensis" \t "https://blast.ncbi.nlm.nih.gov/lnk<@rid@>)* | 97.89 | *Plectosphaerella* sp. | 100.00 | stems | *G. longipes* |
| *Plectosphaerella* sp. GYP-39 | *Plectosphaerella niemeijerarum* | 99.22 | *Plectosphaerella niemeijerarum* | 99.61 | shoot apices | *G. longipes* |
| *Alternaria* sp. GYP-40 | *Alternaria destruens* | 100.00 | *Alternaria* spp.  (*A. alternata*, *A. tenuissima*, etc.) | 100.00 | fibrous roots leaves  main roots | *G. longipes* |
| *Alternaria* sp. GYP-41 | *Alternaria [tectorum](https://www.ncbi.nlm.nih.gov/Taxonomy/Browser/wwwtax.cgi?id=2993421" \o "Taxonomy for Alternaria tectorum" \t "https://blast.ncbi.nlm.nih.gov/lnk<@rid@>)* | 93.28 | *Alternaria* sp. | 100.00 | fibrous roots | *G. longipes* |
| *Botryosphaeria* sp. GYP-42 | *Botryosphaeria [wangensis](https://www.ncbi.nlm.nih.gov/Taxonomy/Browser/wwwtax.cgi?id=2008654" \o "Taxonomy for Botryosphaeria wangensis" \t "https://blast.ncbi.nlm.nih.gov/lnk<@rid@>)* | 99.42 | *Botryosphaeria dothidea* | 99.81 | fibrous roots | *G. longipes* |
| *Botryosphaeria* sp. GYP-43 | *Botryosphaeria [fusispora](https://www.ncbi.nlm.nih.gov/Taxonomy/Browser/wwwtax.cgi?id=1279118" \o "Taxonomy for Botryosphaeria fusispora" \t "https://blast.ncbi.nlm.nih.gov/lnk<@rid@>)* | 94.35 | *Botryosphaeria* spp.  (*B. [dothidea](https://www.ncbi.nlm.nih.gov/Taxonomy/Browser/wwwtax.cgi?command=show&mode=node&id=55169&lvl=)*, *B. neijiangensis*, etc.) | 99.43 | leaves | *G. longipes* |
| *Penicillium* sp. GYP-44 | *Penicillium rubens* | 99.63 | *Penicillium* spp.  (*P. rubens*, *P. chrysogenum*) | 99.82 | fibrous roots shoot apices main roots | *G. longipes* |
| *Stagonosporopsis* sp. GYP-45 | *Stagonosporopsis [dorenboschii](https://www.ncbi.nlm.nih.gov/Taxonomy/Browser/wwwtax.cgi?id=749892" \o "Taxonomy for Stagonosporopsis dorenboschii" \t "https://blast.ncbi.nlm.nih.gov/lnk<@rid@>)* | 99.18 | *Stagonosporopsis cucurbitacearum* | 100.00 | fibrous roots shoot apices main roots stems leaves | *G. longipes* |
| Mortierellaceae sp. GYP-46 | Several Mortierellaceae taxa (*Mortierella*, *Linnemannia*, etc.) | 96.36 | Several Mortierellaceae taxa (*Mortierella*, *Linnemannia*, etc.) | 96.56 | shoot apices | *G. longipes* |
| Mortierellaceae sp. GYP-47 | Several Mortierellaceae taxa (*Mortierella*, *Linnemannia*, etc.) | 99.50 | Several Mortierellaceae taxa (*Mortierella*, *Linnemannia*, etc.) | 100.00 | shoot apices | *G. longipes* |
| *Gibellulopsis* sp. GYP-48 | *Gibellulopsis serrae* | 99.58 | *Gibellulopsis nigrescens* | 100.00 | shoot apices | *G. longipes* |
| *Cercospora* sp. GYP-49 | *Cercospora glycinicola* | 99.80 | *Cercospora* spp.  (*C. chinensis*, *C. canescens*, etc.) | 99.80 | stems | *G. longipes* |
| Botryosphaeriaceae sp. GYP-50 | *Phyllosticta fallopiae* | 99.83 | Several Botryosphaeriaceae taxa  (*Phyllosticta*, *Guignardia*, etc.) | 100.00 | leaves main roots | *G. longipes* |

Note: The “Closest GenBank Matches” were selected based on BLAST results considering both sequence similarity and query coverage. When multiple closely related taxa showed comparable sequence similarity, they were collectively presented using “spp.” or multiple possible matches. For isolates showing high similarity but insufficient resolution among closely related genera or species, assignments were conservatively retained at a higher taxonomic level (e.g., family) to ensure taxonomic accuracy and reliability. These annotations represent BLAST-based comparisons rather than definitive taxonomic identifications.

**Figure S2.** ITS-based phylogenetic analysis of representative endophytic fungal isolates associated with *Gynostemma pentaphyllum* and *Gynostemma longipes*. The phylogenetic tree was constructed based on ITS rDNA sequences using the neighbor-joining (NJ) method with 1000 bootstrap replicates. Bootstrap values greater than 90% are shown at the nodes and marked with green arrows. Branch lengths do not represent evolutionary distances.

**Table S5.** Antibacterial activity of endophytic fungal isolates expressed as inhibition zone diameters (mm).

| **Sample** | ***E. coli*** | | | ***S. aureus*** | | | ***P. aeruginosa*** | | |
| --- | --- | --- | --- | --- | --- | --- | --- | --- | --- |
|  | **10 μL** | **15 μL** | **20 μL** | **10 μL** | **15 μL** | **20 μL** | **10 μL** | **15 μL** | **20 μL** |
| GYP-01 | 19.5 | 21 | 22 | 12.8 | 15 | 17 | 7 | 8.5 | 10 |
| GYP-02 | 14.5 | 15 | 18 | 11.5 | 13.5 | 16 | 6 | 6.5 | 7 |
| GYP-03 | 8 | 10 | 11.5 | 6 | 6.5 | 7 | 8.5 | 10 | 11.5 |
| GYP-04 | 6 | 9 | 11 | 6.5 | 8 | 9 | 8.5 | 10 | 12 |
| GYP-05 | 9 | 11 | 12 | 7.5 | 9 | 10.5 | 10 | 12 | 14 |
| GYP-06 | 8 | 9 | 9.5 | 6 | 6.5 | 7 | 9 | 10.5 | 12 |
| GYP-07 | 8 | 11.5 | 12 | 6 | 6.5 | 8 | 8.5 | 10.5 | 12.5 |
| GYP-08 | 9 | 10.5 | 11.5 | 7 | 9 | 11 | 8.5 | 10.5 | 11.5 |
| GYP-09 | 7.5 | 11 | 13 | 6.5 | 8 | 9 | 10 | 12 | 13.5 |
| GYP-10 | 6 | 18 | 20 | 6 | 7 | 8 | 7 | 8.5 | 10 |
| GYP-11 | 6 | 6 | 8 | 6.5 | 7.5 | 8.5 | 8.5 | 11 | 13 |
| GYP-12 | 6.5 | 7 | 8 | 6 | 7 | 8 | 10 | 12 | 13.5 |
| GYP-13 | 6 | 6 | 6 | 10 | 12 | 14 | 9 | 10 | 12 |
| GYP-14 | 6.5 | 6 | 18.5 | 10.5 | 12.5 | 14 | 9 | 11 | 14 |
| GYP-15 | 10.5 | 12 | 13.5 | 10.5 | 12 | 13.5 | 9 | 10.5 | 13 |
| GYP-16 | 10 | 14 | 16 | 10 | 12 | 14 | 7.5 | 9 | 11 |
| GYP-17 | 7 | 9 | 10 | 9.8 | 11.5 | 13.5 | 8.5 | 10 | 11.5 |
| GYP-18 | 6 | 6 | 7 | 12.5 | 15 | 17 | 12 | 14.5 | 17 |
| GYP-19 | 7.5 | 10 | 12 | 14.3 | 16 | 18 | 7.5 | 9 | 11.5 |
| GYP-20 | 6 | 6 | 8 | 12 | 14 | 16 | 10.5 | 12.5 | 14 |
| GYP-21 | 8.5 | 8.5 | 9.5 | 12.8 | 14.5 | 16.5 | 8 | 10 | 11 |
| GYP-22 | 6 | 6.5 | 8 | 11.5 | 12 | 14 | 8.5 | 10 | 12 |
| GYP-23 | 6.5 | 7 | 8 | 6 | 7 | 8 | 7.5 | 9 | 10.5 |
| GYP-24 | 6.5 | 7.5 | 8 | 6 | 7 | 8.5 | 8 | 9.5 | 11 |
| GYP-25 | 6 | 8 | 10 | 6.5 | 8 | 10 | 7.5 | 9 | 11 |
| GYP-26 | 6 | 8.5 | 10 | 6 | 6 | 7.5 | 7.5 | 9 | 10.5 |
| GYP-27 | 9 | 12.5 | 19 | 7.5 | 9 | 10 | 8.5 | 10.5 | 12 |
| GYP-28 | 16 | 19 | 21 | 9 | 11 | 13.5 | 9 | 10.5 | 12 |
| GYP-29 | 7 | 6 | 6 | 6 | 7 | 7.5 | 10 | 12 | 13.5 |
| GYP-30 | 7 | 10 | 11.5 | 6.5 | 8 | 9 | 7 | 8.5 | 10 |
| GYP-31 | 9.5 | 12 | 15 | 8 | 9 | 10 | 9 | 11 | 12 |
| GYP-32 | 9 | 11 | 12 | 9 | 11 | 13 | 9 | 10.5 | 12 |
| GYP-33 | 8.5 | 10.5 | 12 | 6 | 7 | 8 | 8 | 9.5 | 11 |
| GYP-34 | 9.5 | 11 | 12.5 | 8.5 | 9 | 11 | 6 | 6.5 | 7 |
| GYP-35 | 11.5 | 14 | 16 | 9.5 | 13 | 15.5 | 8 | 9.5 | 11 |
| GYP-36 | 25.5 | 29 | 31 | 23.3 | 25 | 27 | 8.5 | 10 | 11.5 |
| GYP-37 | 9 | 10 | 10.5 | 6 | 6.5 | 7 | 6.5 | 7.5 | 9 |
| GYP-38 | 6 | 6 | 6 | 6 | 7 | 8 | 6 | 7 | 8.5 |
| GYP-39 | 10.5 | 12 | 13 | 10 | 12 | 13.5 | 8 | 9.5 | 11 |
| GYP-40 | 6.5 | 13.5 | 15 | 9 | 10.5 | 12 | 8 | 10 | 11 |
| GYP-41 | 12 | 12.5 | 13 | 8.5 | 11 | 12 | 9 | 11 | 12 |
| GYP-42 | 6.5 | 7.5 | 8.5 | 8.5 | 10.5 | 12 | 9 | 10.5 | 12.5 |
| GYP-43 | 6 | 6 | 7 | 9 | 10.5 | 12 | 7 | 9 | 10.5 |
| GYP-44 | 7 | 8.5 | 10 | 7.5 | 9 | 10.5 | 8 | 9.5 | 10.5 |
| GYP-45 | 6.5 | 7.5 | 8 | 9 | 10.5 | 12 | 6 | 6 | 7 |
| GYP-46 | 8 | 9.5 | 12 | 9.2 | 11 | 12.5 | 9.5 | 12 | 13.5 |
| GYP-47 | 6 | 9 | 10 | 9 | 10.5 | 12 | 6 | 6.5 | 7 |
| GYP-48 | 8 | 11.5 | 12 | 7 | 8.5 | 10 | 10.5 | 13 | 15.5 |
| GYP-49 | 9 | 10.5 | 13.5 | 7.2 | 9 | 11 | 8.5 | 10 | 12.5 |
| GYP-50 | 10.5 | 13.5 | 16 | 11 | 13 | 14.5 | 8 | 9.5 | 12 |
| penicillin sodium, 10 µg/disc | 35 | | | 21.4 | | | 9.5 | | |
| vancomycin, 30 µg/disc | 30 | | | 26.5 | | | 9 | | |
| gentamicin, 10 µg/disc | 36.5 | | | 24.5 | | | 19 | | |

Note: Values represent mean inhibition zone diameters (mm) of three independent replicates. Statistical comparisons among application volumes (10, 15, and 20 μL) were performed using one-way ANOVA. Significant differences were observed between application volumes (p < 0.05).

**Figure S3.** Principal component analysis (PCA) of antibacterial activity profiles of endophytic fungal isolates grouped by (a) host species and (b) tissue origin. PCA was performed based on standardized inhibition zone diameters against Escherichia coli, Staphylococcus aureus, and Pseudomonas aeruginosa. Each point represents an individual fungal isolate. Ellipses represent 95% confidence intervals and are shown for visualization purposes only. PERMANOVA analysis indicated that neither host species (Pseudo-F = 1.1589, P = 0.307) nor tissue origin (Pseudo-F = 0.3801, P = 0.879) had statistically significant effects on antibacterial activity profiles.

**Figure S4.** Hierarchical clustering heatmap of antibacterial activity profiles of endophytic fungal isolates based on raw inhibition zone diameters. The heatmap was constructed using inhibition zone diameters (mm) against *Escherichia coli*, *Staphylococcus aureus*, and *Pseudomonas aeruginosa* at three application volumes (10, 15, and 20 μL). Hierarchical clustering was performed using Euclidean distance and Ward’s linkage method. The color scale represents inhibition zone diameters (mm), with darker colors indicating larger inhibition zones. Rows represent individual fungal isolates, and columns represent bacterial species and application volumes.
